# Prognostic Value of Pretreatment Neutrophil-to-Lymphocyte Ratio in HER2-Positive Metastatic Breast Cancer

**DOI:** 10.3390/curroncol29090483

**Published:** 2022-08-25

**Authors:** Bin Shao, Xiaoran Liu, Huiping Li, Guohong Song, Lijun Di, Hanfang Jiang, Ying Yan, Ruyan Zhang, Ran Ran, Jiayang Zhang, Yaxin Liu, Huan Wang, Jing Wang

**Affiliations:** Key Laboratory of Carcinogenesis and Translational Research (Ministry of Education/Beijing), Department of Breast Oncology, Peking University Cancer Hospital & Institute, Beijing 100142, China

**Keywords:** neutrophil-to-lymphocyte ratio, HER2 positive, metastatic breast cancer, prognosis, anti-HER2 treatment

## Abstract

This study aimed to examine the prognostic value of the neutrophil-to-lymphocyte ratio (NLR) and other clinicopathological features in HER2+ MBC patients who received first-line anti-HER2 therapy. A total of 129 patients were assigned to NLR-low and NLR-high groups based on a cutoff value of 3.0 at baseline. Peripheral blood lymphocyte subsets and gene mutations in circulating tumor DNA were analyzed by flow cytometry and Next-generation sequencing, respectively. Survival was evaluated by the Kaplan–Meier method and Cox regression analysis. Of the 129 patients, 77 and 52 were assigned to the NLR-low (≤3) and NLR-high (>3) groups, respectively. Compared with NLR-high patients, the NLR-low patients had significantly longer median progression-free survival (PFS) (11.7 vs. 7.7 months) (*p* = 0.001, HR = 2.703 95% CI 1.543–4.736 and overall survival (OS) (37.4 vs. 28.7 months) (*p* = 0.044, HR = 2.254 95% CI 1.024–4.924). Furthermore, this association was independent of metastatic sites or estrogen receptor status. Peripheral blood CD3+ (*p* = 0.034) and CD4+ (*p* = 0.010) T cell numbers were significantly higher in the NLR-low group than the NLR-high group. The mutational profile of MBC was generally similar between the two groups. Baseline NLR was a prognostic factor of PFS and OS for patients with HER2+ MBC in the first-line setting. These results may facilitate the selection of patients who will benefit most from anti-HER2 treatment.

## 1. Introduction

Breast cancer has become the most common cancer in the world. In 2020, there were an estimated 2.3 million newly diagnosed cases, accounting for 11.7% of all cancers. Despite recent improvements in treatment strategies, breast cancer causes approximately 685,000 deaths annually, making it the fifth leading cause of cancer mortality [1]. Breast cancer is a heterogeneous disease with multiple subtypes that differ markedly in their biology and prognosis [2,3]. The proportion of human epidermal growth factor receptor 2 positive (HER2+) breast cancer is about 20–30%. Before anti-HER2 targeted therapy, HER2+ was considered a poor prognostic factor [4].

Age, tumor stage, grade, and hormone receptor status are among the most important prognostic factors for patients with metastatic breast cancer (MBC), which is a multifactorial disease with complex biological behavior. A consensus-validated life expectancy model for MBC has not yet been developed [5]. Previous studies have shown that the immune system plays an important role in anti-HER2 targeted therapy [6]. Among them, antibody-dependent cytotoxicity (ADCC) mediated by natural killer (NK) cells and granulocytes is the main mechanism of trastuzumab (anti-HER2 monoclonal antibody) [7]. Additionally, the abundance of tumor-infiltrating lymphocytes (TILs) significantly correlates with the prognosis of trastuzumab-treated patients with MBC [8], further supporting an important interaction between host immunity and anti-HER2 therapy. The North Central Cancer Treatment Group trial N9831 demonstrated that enriched immune function gene expression in the tumor was a predictor of relapse-free survival in MBC patients receiving chemotherapy with trastuzumab but not in those receiving chemotherapy alone [9]. Preclinical studies have indicated that ADCC contributes to the anti-HER2+ tumor effects of trastuzumab [10]; However, there are few data on the prognostic role of immune cells for survival in HER2-positive breast cancer patients [11].

Neutrophils and lymphocytes are important types of anti-tumor immunity. More and more studies have investigated the prognostic value of neutrophils and lymphocytes in various cancer types, including breast cancer, especially the ratio of neutrophils to lymphocytes (NLR) [12,13]. However, few studies have examined the prognostic significance of NLR in patients, specifically with HER2+ breast cancer [14]. Ulas et al. reported that there was no significant association between disease-free survival (DFS) or overall survival (OS) with NLR in patients with early breast cancer who received adjuvant trastuzumab [15]. While Yao et al. found that NLR has a significant prognostic value regardless of molecular subtype [16]. Thus, additional studies are required before the prognostic value of NLR in HER2+ breast cancer can be clarified.

In the present study, we investigated the relationship between baseline NLR and survival in patients with HER2+ MBC who received trastuzumab as first-line therapy. We also examined the relationship between NLR and various clinicopathologic factors, including the abundance of circulating lymphocyte subsets and gene mutations present in circulating tumor DNA.

## 2. Methods

### 2.1. Study Design

We retrospectively identified 129 consecutive female patients who were diagnosed with HER2+ MBC and received first-line anti-HER2-based treatment at Peking University Cancer Hospital between January 2015 and June 2020. All patients had histologically confirmed invasive HER2+ breast cancer. HER2 positivity was centrally confirmed by using an immunohistochemical (IHC) score of 3+ or, for those with an IHC score of 2+, a positive fluorescence in situ hybridization test, in conformity with the American Society of Clinical Oncology/College of American Pathologists HER2 testing in breast cancer guidelines [17].

Exclusion criteria included concurrent infectious disease, autoimmune disease, hematological disorder, or other malignancies; insufficient clinical data; or withdrawal after the first cycle. Treatment efficacy was evaluated using Response Evaluation Criteria in Solid Tumors (RECIST) version 1.1 [18]. Overall response rate (ORR) was defined as the proportion of patients with a complete response (CR) or partial response (PR) to therapy. Clinical benefit rate (CBR) was defined as the percentage of patients with CR, PR, or stable disease (SD). Progression-free survival (PFS) was defined as the time from treatment initiation to disease progression, death, or loss to follow-up. OS was defined as the time from treatment initiation to death or loss to follow-up.

This study was approved by the Peking University Cancer Hospital Ethical Committee (No. 2016KT47) and was conducted in accordance with the Declaration of Helsinki. Informed consent was waived by the committee because of the retrospective nature of the study. 

### 2.2. Measurement of NLR

Baseline NLR was calculated as the neutrophil count divided by the lymphocyte count in blood samples taken within 7 days before initiation of trastuzumab treatment. Previous work indicated that the NLR range can be large (2.5–4.0), and a standard cutoff value has not yet been agreed upon [19]. In this study, the median value of NLR is 2.87 (range 0.02–14.33), and the mean value was 3.08 ± 2.15. We adopted 3.0 as the cutoff value for the assignment of patients to NLR-low and NLR-high groups based on the value reported by previous studies [20].

### 2.3. Detection of Peripheral Blood T Lymphocyte Subsets

Peripheral blood samples (200 μL) were incubated with anti-human antibody cocktails in the dark for 10 min at room temperature, and samples were then subjected to hemolysis for an additional 10 min. The cells were centrifuged for 5 min at 1300× *g* at room temperature and resuspended in 500 μL phosphate-buffered saline for flow cytometry. Antibody cocktails included CD3-PC5/CD4-FITC/CD8-PE (IM1650), CD3-FITC/CD16/CD56-PE (A07735), CD14/16-FITC/CD85k (ILT3)-PE/CD33-PC5 (A23413), CD4-FITC (A007750), CD8-FITC (A07756), CD19-PC5 (A07771), and CD25-PE (A07774) (all from Beckman Coulter, Brea, CA, USA). Flow cytometry was performed using an FC500 and CXP analysis software (both Beckman Coulter). Each analysis included 10,000 gated events.

### 2.4. Extraction of ctDNA

Cell-free DNA (cfDNA), which includes ctDNA, was extracted from cell-free plasma samples, and gDNA was extracted from purified peripheral blood mononuclear cells (PBMCs). Of the 129 patients, 57 had baseline peripheral blood samples available for cfDNA extraction, and 57 matched PBMC samples were available for gDNA extraction. Briefly, blood samples were centrifuged at 820× *g* for 10 min, the supernatants were transferred to fresh tubes and re-centrifuged at 16,000× *g* for 10 min, and the supernatants were stored at −80 °C. cfDNA was extracted using a QIAamp circulating nucleic acid kit (Qiagen, Germantown, MD), and the quantity and quality were checked using a Qubit fluorimeter and Bioanalyzer 2100. Samples with severe gDNA contamination were further processed using bead-based size selection to remove large gDNA fragments. gDNA was extracted from matched PBMC samples. Up to 250 ng gDNA was enzymatically fragmented and stored at −20 °C. Both cfDNA and gDNA were quantified using a LINE1 qPCR assay [21].

### 2.5. Library Preparation, Capture, and Sequencing

Extracted cfDNA (5–30 ng) or fragmented gDNA (40 ng) were subjected to library construction, including end-repair dA-tailing and adapter ligation. Ligated library fragments with appropriate adapters were amplified by PCR. The amplified DNA libraries were further evaluated using a Bioanalyzer 2100, and samples with sufficient material proceeded to hybrid capture. Library capture was conducted using biotin-labeled DNA probes. In brief, the library was hybridized overnight with the PredicineCARE^TM^ 152-gene panel reagents (Huidu Shanghai Medical Sciences Ltd., Shanghai, China) (Supplementary Table S1) and captured with beads. The unbound fragments were washed away, and the enriched fragments were amplified by PCR. Purified products were checked on a Bioanalyzer 2100 and sequenced using a HiSeq X Ten (Illumina, San Diego, CA, USA) and a paired-end 2 × 150 bp sequencing kit (Illumina).

### 2.6. Statistical Analysis

Statistical tests were selected based on the distribution of variables. Student’s *t*-tests were used to compare normally distributed variables, and Mann–Whitney U tests were used for non-normally distributed variables. The chi-square test or Fisher’s exact test, as appropriate, was performed to compare clinical and pathological characteristics between two groups stratified by the NLR value (NLR ≤ 3 vs. NLR > 3). Survival curves were obtained using the Kaplan–Meier method and compared using a log-rank test. Univariable and multivariable Cox regression analysis was performed to identify factors that independently influenced survival. The patients’ age, primary breast cancer stage, histological grade, hormone receptor status, disease-free survival, visceral metastasis, number of metastatic sites, and NLR were included in the multivariable analysis. All tests were two-tailed, and *p* < 0.05 was considered statistically significant. SPSS^®^ software version 22 (IBM Inc., Broadway, NY, USA) was used for the statistical analysis.

## 3. Results

### 3.1. Patient Characteristics

A total of 129 patients with HER2+ MBC who received at least two cycles of anti-HER2 targeted treatment as first-line therapy were enrolled in this study. All the patients could evaluate the response rate. Of those, 16 were lost to follow-up, and 50 died. The median follow-up time was 21.0 months (range 2.0–46.0 months). The patients’ clinicopathological characteristics are presented in Table 1. The median age at diagnosis was 51.0 years (range 25–82 years), 63 (48.8%) patients were hormone-receptor-positive, and 89 (69%) patients had visceral metastasis. The median value of NLR is 2.87 (range 0.02–14.33). In total, 77 and 52 patients were assigned to the NLR-low (≤3) and NLR-high (>3) groups, respectively. The two NLR groups had similar clinicopathological features (Table 1).

### 3.2. Association between NLR and Response to First-Line Anti-HER2 Treatment

**Figure 1:**
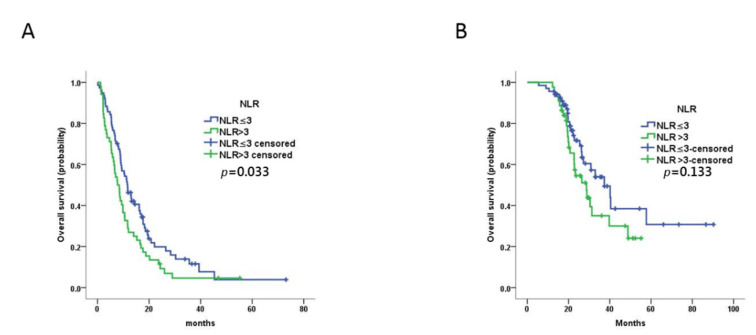
Prognostic value of baseline NLR in MBC patients treated with trastuzumab as first-line therapy. (**A**,**B**) Kaplan–Meier plots of progression-free survival (**A**) and overall survival (**B**) for patients assigned to NLR-high (*n* = 52) and NLR-low (*n* = 77) groups at baseline.

The ORR was 48.8% (63/129), CBR was 82.9% (107/129), PR was 48.8% (63/129), SD was 34.1% (44/129), and progressive disease (PD) was 17.1% (22/129). The ORR and CBR were not significantly different between the NLR-low and NLR-high groups (*p* = 0.828 and *p* = 0.309, respectively; Table 2). The median PFS of all patients was 9.9 months (95% confidence interval (CI) 8.0–11.7 months). The median PFS for the NLR-low group (11.7 months, 95% CI 8.9–14.4 months) was significantly longer than that for the NLR-high group (7.7 months, 95% CI 5.5–10.0 months; *p* = 0.033) (Figure 1). Univariable analysis revealed that PFS was not associated with any of the other variables evaluated (age at cancer diagnosis, primary breast cancer stage, grade, hormone receptor expression, DFS, visceral metastasis, number of metastatic sites). Multivariable analysis confirmed the prognostic value of NLR (*p* = 0.001, hazard ratio (HR) = 2.703 95% CI 1.543–4.736) (Table 3).

The median OS for all patients was 30.8 months (95% CI 22.0–39.6 months). In contrast to PFS, there was no significant difference between the OS of the NLR-low group (37.4 months, 95% CI 25.7–49.0 months) and the NLR-high group (28.7 months, 95% CI 21.4–36.1 months) in univariable analysis (*p* = 0.133) (Figure 1). However, NLR (*p* = 0.044, HR = 2.254 95% CI 1.024–4.924) and pathologic grade (*p* = 0.023, HR = 2.712 95% CI 1.149–6.402) were prognostic factors for OS in multivariable analysis (Table 4).

### 3.3. Association between NLR and Peripheral Blood T Lymphocyte Subsets

We previously showed that the abundance of peripheral blood CD8 + CD28+ cytotoxic T lymphocytes is predictive of PFS in breast cancer, particularly in patients with HER2+ breast cancer who received anti-HER2 therapy [22]. Therefore, we compared the distribution of several peripheral blood lymphocyte subsets between the NLR-low and NLR-high groups. Patients in the NLR-low had a significantly higher percentage of CD3+ T cells (*p* = 0.034) and CD4+ T cells (*p* = 0.010) than patients in the NLR-high group. However, the abundance of the remaining lymphocyte subsets, including CD8+ CD28+ cells, did not differ significantly between the groups (Table 5).

### 3.4. Association between NLR and Gene Mutations

**Figure 2:**
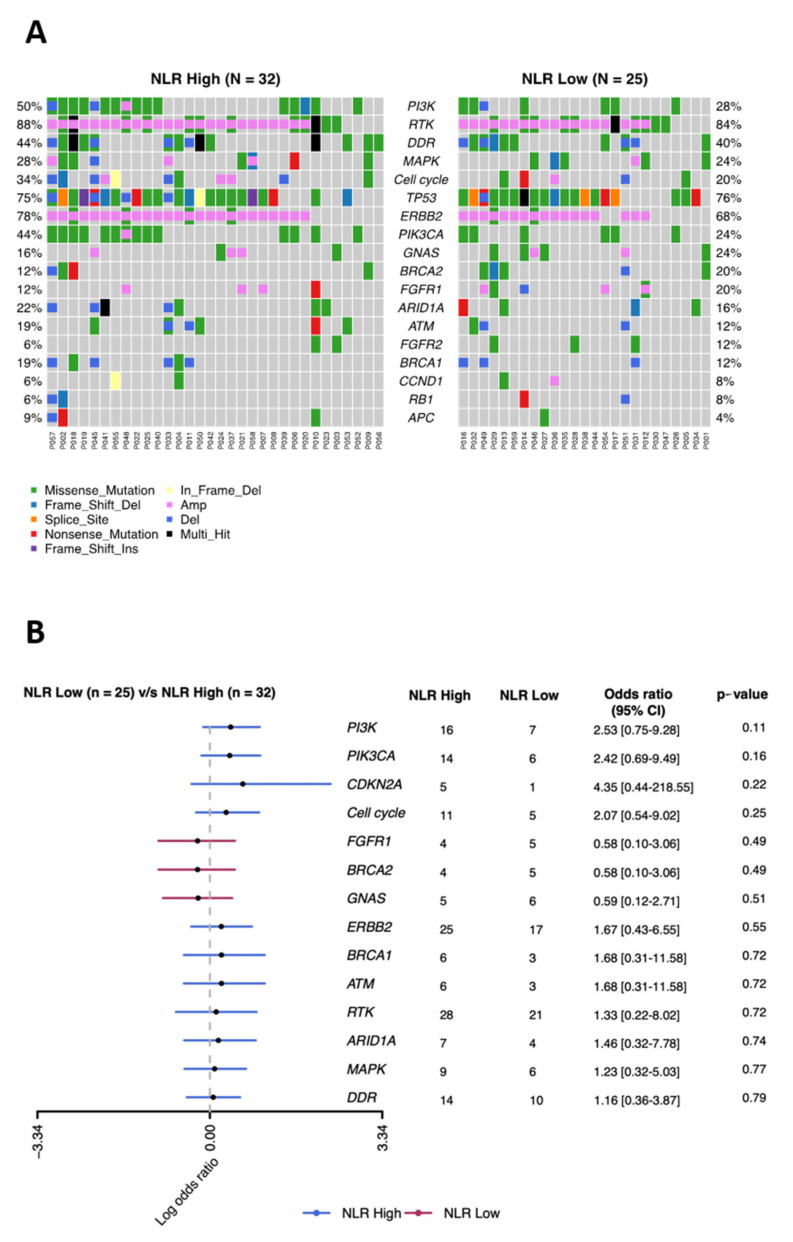
Comparison of the mutational landscape of 152 tumor-related genes in MBC patients with low and high baseline NLR. (**A**) Distribution of single nucleotide variants and copy number variants in the indicated genes with mutation rates of genes ≥5%. (**B**) Relative enrichment of key genes in the NLR-high versus the NLR-low group.

To determine whether genomic alterations in MBC tumors correlated with patient NLR and to identify potential genomic markers related to NLR, we performed a PredicineCARE screen of plasma cfDNA from 59 patients. This screen analyzes 152 cancer-related genes by next-generation sequencing and has broad genomic coverage. Overall, the genomic profile of cfDNA from the NLR-low and NLR-high groups were similar, with frequent mutations identified in components of the phosphoinositide 3-kinase (PI3K) signaling pathway (AKT1, AKT2, MTOR, PIK3CA, PIK3CB, PIK3CD, PIK3R1, PTEN, RHEB, STK11, TSC1, and TSC2) (50% in the NLR-low group vs. 28% in the NLR-high group), receptor tyrosine kinases (ABL1, EGFR, ERBB2, ERBB3, ERBB4, PDGFRA, PDGFRB, MET, FGFR1, FGFR2, FGFR3, FGFR4, FLT3, ALK, RET, ROS1, KIT, IGF1R, NTRK1, NTRK2, and NTRK3) (88% vs. 84%), DNA damage repair pathway (ATM, ATR, BRCA1, BRCA2, CHEK2, MLH1, MSH2, MSH6, MUTYH, PALB2, PMS2, POLD1, POLE, RAD51B, RAD51C, RAD51D, XPC, and XRCC1) (44% vs. 40%), mitogen activated protein kinase (MAPK) signaling pathway (PTPN11, KRAS, HRAS, NRAS, RIT1, ARAF, BRAF, RAF1, MAP2K1, MAP2K2, MAPK1, and NF1) (28% vs. 24%), and cell cycle pathway (CDKN2A, CDKN2B, CCND1, CCND2, CCND3, CCNE1, CDK4, CDK6, RB1, and TOP2A) (34% vs. 20%) (Figure 2A,B). The pathway mutation was defined as mutated if any of the genes in the given pathway was mutated. Although none of the mutation frequencies differed significantly between the NLR-low and NLR-high groups, several mutations were slightly more common in the NLR-high group; these included genes in the PI3K pathway (odds ratio (OR) = 2.53) and cell cycle pathway (OR = 2.07), as well as the *PIK3CA*, which encodes PI3K catalytic subunit alpha (OR = 2.42), and *CDKN2A*, which encodes cyclin-dependent kinase inhibitor 2A (OR = 4.35) (Figure 2B).

## 4. Discussion

This study demonstrated that high pretreatment NLR was associated with significantly worse prognosis in HER2+ MBC patients receiving first-line anti-HER2 therapy compared with patients with low NLR. Patients with high baseline NLR also had lower baseline CD3+ T cell and CD4+ T cell levels; however, there were no significant differences between the two groups with respect to variation in sequence or copy number of 152 genes with relevance to cancer.

### 4.1. NLR and Survival

NLR has a predictive prognostic value in some solid malignant tumors [23], and baseline NLR measured prior to treatment initiation can predict the survival of patients with early breast cancer [13,20,24]. A meta-analysis of eight studies showed that elevated NLR was associated with a significantly lower overall survival in patients with breast cancer [19]. Previous studies have also shown a significant correlation between NLR and survival in patients with MBC [25,26,27,28,29,30]. However, we consider that the results of those studies should be interpreted with caution because not all molecular subtypes of breast cancer were represented, and the heterogeneity of the study populations was very large.

### 4.2. NLR and HER2+ Breast Cancer Subtypes

The predictive value of NLR in breast cancer of different molecular subtypes is controversial. Some studies have shown that high NLR is significantly correlated with poor prognosis in triple-negative breast cancer (TNBC) but not in luminal A-like, luminal B-like, or HER2-enriched subtypes [8]. The predictive prognostic value of TILs has been established for TNBC [31], but whether peripheral blood neutrophils or lymphocytes have similar value in TNBC is not known. Noh et al. [13] found a significant prognostic value of NLR in luminal subtype breast cancer (ER+ or PR+, HER2−), whereas Yao et al. [16] found that NLR has a significant prognostic value regardless of luminal type or TNBC. Therefore, the prognostic significance of NLR in HER2+ breast cancers remains unclear.

Different subtypes of breast cancer are associated with immune heterogeneity. TNBC and HER2+ breast cancers with aggressive biological behavior have high genomic instability and tumor mutation burden, both of which promote the production of tumor neoantigens and increase antitumor immune activity. In addition, HER2 itself acts as a tumor-associated neoantigen in HER2+ breast cancer. Cytotoxic therapy and anti-HER2 targeted therapy can further activate the immune system through immunogenic cell death and ADCC, respectively [6]. The antitumor effects of anti-HER2 antibodies are mediated, at least in part, through ADCC by innate immune cells [18]. The binding of anti-HER2 antibodies to HER2 induces NK cell-mediated ADCC, which is followed by induction of tumor-specific cytotoxicity mediated by cells of the adaptive immune system [32].

Of the two previous studies examining NLR and HER2+ breast cancer [30,33], one found that NLR correlated with PFS and OS in patients who received T-DM1 [30], and the second study found that improved PFS was not significantly associated with NLR but absolute lymphocyte count ≥1500/μL. In the present study, we also found that NLR was an independent predictor of PFS and OS in patients with HER2+ MBC undergoing first-line anti-HER2 therapy. Therefore, further study of NLR in breast cancer patients is warranted, especially studies of different treatment lines and drugs.

### 4.3. Optimal Cutoff Point of the NLR

The optimal NLR for determining associations with survival in patients with HER2-positive MBC is still unclear. Azab et al. [34] found no significant difference between the three lower quartiles of NLR and mortality in patients with breast cancer, suggesting that there may be a threshold value at which NLR correlates with mortality. In the past, receiver operating characteristic curve analysis has been used to determine the optimal NLR value for predicting survival, but no consensus value has yet been identified [13,16]. In the present study, with a limited number of subjects, we selected 3.0 as the cutoff value based on a previous study [20].

### 4.4. NLR and Lymphocyte Subsets

We previously showed that circulating peripheral CD8+ CD28+ T cell ratio could predict PFS in HER2+ MBC patients receiving anti-HER2 therapy (13.1 vs. 5.6 months, *p* = 0.001) [22], prompting us to further explore the relationship between NLR and the abundance of additional peripheral blood lymphocyte subsets in the present study. We found that NLR-low patients had higher levels of peripheral CD3+ T cells and CD4+ T cells but not of any other lymphocyte subsets examined compared with NLR-high patients.

T cells play a crucial role in the antitumor immune response, and the level of tumor-infiltrating T cells has been correlated with patient survival in some cancers [35], and CD4+ T cells are generally mediated antitumor effects by regulating the activity of other cells by cytokine production, and by establishing long-term antitumor memory [36]. CD4+ T cells can activate monocytes/macrophages, NK cells, and specific CD8+ cytotoxic T cells and are thus pivotal to the antitumor response, suggesting an explanation for the significant association between circulating CD4+ cells and OS previously demonstrated in some cancers [37]. In addition, the HER2-specific response of circulating CD4+ T cells after anti-HER2 treatment has been correlated with HER2+ breast cancer recurrence. An anti-HER2 response could be detected in vitro by measuring the production of interferon-γ (IFN–γ), interleukin (IL)-4, and IL-10 after stimulation of PBMCs with HER2 peptides (REFS?). Datta J et al. found that patients with an anti-HER2 CD4+ T cell response had a higher DFS rate than those lacking a CD4+ T cell response [38]. Our finding of elevated CD4+ T cells in NLR-low patients, who also had a longer median PFS compared with NLR-high patients, is thus consistent with the possibility that NLR-low patients may have mounted a more robust antitumor response than NLR-high patients.

### 4.5. NLR and ctDNA

Analysis of ctDNA has proven to be useful not only for examining the genomic status of tumors but also for shedding light on other aspects of the disease, including the immune status. Pedersen et al. [39] reported that the baseline ctDNA level was closely related to the therapeutic effect of immune checkpoint inhibitors in patients with metastatic melanoma. DNA can stimulate the immune response via regulation of IFN and other proinflammatory mediators in immune cells in a manner dependent on structure and sequence (REFS), and immune cell activation can be observed with DNA alone or in complex with other molecules [40]. The large number of nucleic acid receptors expressed by immune cells attests to the important role of DNA in the innate immune system [41]. In our study, however, we found no significant genomic differences between ctDNA from the NLR-high and -low patient groups. However, several signaling and cell cycle pathway genes were slightly more frequently mutated in the NLR-high group, suggesting that additional investigations with a larger sample size will be of interest.

### 4.6. Limitations

First, this was a retrospective study, and the main limitations are retrospective data collection and the single-center design. Second, the sample size was relatively small for survival analysis. Third, the critical cutoff value for the NLR was based on findings from a previous study. Larger-scale confirmatory studies will be needed to validate the predictive role of NLR in HER2+ MBC patients undergoing first-line anti-HER2 therapy. Finally, we analyzed the data employing a single baseline NLR value, and it is possible that dynamic monitoring of NLR throughout treatment may be helpful in determining its predictive value.

## 5. Conclusions

Our results suggest that baseline NLR, an easily measured biomarker, is an independent predictor of PFS and OS for patients with HER2+ MBC undergoing first-line anti-HER2 treatment. NLR-low status in this patient population at baseline may reflect an enhanced activity of the immune system. These findings are a useful reminder that NLR should be taken into consideration when making treatment decisions.

## Figures and Tables

**Table 1:** Clinicopathological characteristics of 129 patients with HER2+ MBC.

Characteristics	All		NLR ≤ 3 (*n* = 77)		NLR > 3 (*n* = 52)		*p*
	*n*	%	*n*	%	*n*	%	
Age of diagnosis (years)							
≤50	59	45.7	33	42.9	26	50.0	0.424
>50	70	54.3	44	57.1	26	50.0	
Histology							
IDC	124	96.1	74	96.1	50	96.2	1.000 *
ILC	4	3.1	2	2.6	2	3.8	
Others	1	0.8	1	1.3	0	0.0	
Primary breast cancer stage							
I	11	8.5	4	5.2	7	13.5	0.222 *
II	43	33.3	24	31.2	19	36.5	
III	31	24.0	22	28.6	9	17.3	
IV	33	25.6	18	23.4	15	28.8	
Unknown	11	8.5	9	11.7	2	3.8	
Grade							
1 or 2	82	63.6	48	62.3	34	65.4	0.747
3	34	26.4	21	27.3	13	25.0	
Unknown	13	10.1	8	10.4	5	9.6	
HR expression							
Positive	63	48.8	38	49.4	25	48.1	0.887
Negative	66	51.2	39	50.6	27	51.9	
DFS							
≤2 years	29	30.2	17	28.8	12	32.4	0.707
>2 years	67	69.8	42	71.2	25	67.6	
Visceral metastasis							
Yes	89	69.0	51	66.2	38	73.1	0.410
No	40	31.0	26	33.8	14	26.9	
Number of metastatic sites							
≤2	88	68.2	14	18.2	31	59.6	0.085
>2	41	31.8	63	81.8	21	40.4	
Therapy							
Trastuzumab	117	87.6	68	88.3	49	94.2	0.677 *
Trastuzumab plus persuzumab	4	3.1	2	2.6	2	3.8	
TKI	10	7.8	7	9.1	3	5.8	
T-DM1	2	1.6	2	2.6	0	0.0	
First-line therapy							
Chemotherapy	121	93.8	69	89.6	52	100.0	0.043 *
Endocrine	6	4.7	6	7.8	0	0.0	
T-DM1	2	1.5	2	2.6	0	0.0	

Note: DFS, disease-free survival (the time from surgery of the primary tumor to recurrence); HR, hormone-receptor-positive (estrogen receptor (ER) ≥ 10% and/or progesterone receptor (PR) ≥ 10%); IDC, invasive ductal carcinoma; ILC, invasive lobular carcinoma; MBC, metastatic breast cancer; NLR, neutrophil-to-lymphocyte ratio; TKI, tyrokinase inhibitor; T-DMI, trastuzumab emtansine. * *p* values determined by Fisher’s exact test; all other *p* values determined by Chi-squared test.

**Table 2:** Correlations between baseline NLR and treatment outcomes in patients with MBC.

	All		NLR ≤ 3 (*n* = 77)		NLR >3 (*n* = 52)		*p*
	*n*	%	*n*	%	*n*	%	
PR	63	48.8	37	48.1	26	50.0	0.452
SD	44	34.1	29	37.7	15	28.8	
PD	22	17.1	11	14.3	11	21.2	
ORR (CR + PR)	63	48.8	37	48.1	26	50.0	0.828
CBR (CR + PR + SD)	107	82.9	66	85.7	41	78.8	0.309
PFS (months)	9.9		11.7 (95%CI 8.0–11.7)		7.7 (95%CI 5.5–10.0)		0.033
OS (months)	30.8		37.4 (95%CI 25.7–49.0)		28.7 (95%CI 21.4–36.1)		0.133

Note: CBR, clinical benefit rate; CR, complete response; MBC, metastatic breast cancer; NLR, neutrophil-to-lymphocyte ratio; ORR, overall response rate; OS, overall survival; PR, partial response; PD, progressive disease; PFS, progression-free survival; SD, stable disease. In total, 16 patients were lost to follow-up.

**Table 3:** Univariable and multivariable analysis of progression-free survival.

Characteristics	*n*	Median PFS (Moths)	Univariable Analysis		Multivariable Analysis	
			*p*	HR	*p*	HR
Age of diagnosis (years)						
≤50	59	10.6	0.409	0.854 (0.587–1.243)	0.302	0.751 (0.435–1.294)
>50	70	9.4				
Primary breast cancer stage						
I	11	11.7	0.157	1.177 (0.972–1.426)	0.165	1.401 (0.871–2.254)
II	43	10.6				
III	31	8.8				
IV	33	9.9				
Grade						
1 or 2	82	9.9	0.222	1.314 (0.846–2.040)	0.244	1.434 (0.782–2.628)
3	34	8.4				
HR expression						
Positive	63	10.6	0.990	0.998 (0.684–1.456)	0.603	1.174 (0.641–2.153)
Negative	66	8.6				
DFS						
≤2 years	88	7.6	0.076	0.656 (0.410–1.050)	0.118	0.596 (0.312–1.139)
>2 years	41	11.7				
Visceral metastasis						
Yes	89	9.4	0.903	1.025 (0.687–1.530)	0.675	0.879 (0.479–1.610)
No	40	9.9				
Number of metastatic sites						
≤2	88	9.1	0.677	1.088 (0.731–1.618)	0.625	0.861 (0.472–1.570)
>2	41	10.5				
NLR						
≤3	77	11.7	0.033	1.500 (1.031–2.182)	0.001	2.703 (1.543–4.736)
>3	52	7.7				

Note: DFS, disease-free survival (the time from surgery of the primary tumor to recurrence); HR, hormone-receptor-positive (estrogen receptor (ER) ≥ 10% and/or progesterone receptor (PR) ≥ 10%); NLR, neutrophil-to-lymphocyte ratio.

**Table 4:** Univariable and multivariable analysis of overall survival.

Characteristics	*n*	Median PFS (Moths)	Univariable Analysis		Multivariable Analysis	
			*p*	HR	*p*	HR
Age of diagnosis (years)						
≤50	51	37.4	0.282	1.368 (0.771–2.425)	0.475	1.342 (1.599–3.004)
>50	62	28.9				
Primary breast cancer stage						
I	8	48.8	0.088	0.933 (0.697–1.248)	0.198	1.577 (0.788–3.155)
II	41	28.9				
III	27	25.4				
IV	27	40.2				
Grade						
1 or 2	72	39.7	0.145	1.623 (0.841–3.131)	0.023	2.712 (1.149–6.402)
3	31	26.3				
HR expression						
Positive	57	30.8	0.442	0.801 (0.454–1.413)	0.627	1.243 (0.517–2.986)
Negative	56	31.3				
DFS						
≤2 years	25	21.0	0.024	0.479 (0.249–0.919)	0.075	0.489 (0.223–1.075)
>2 years	61	30.8				
Visceral metastasis						
Yes	35	31.3	0.572	1.190 (1.650–2.181)	0.251	1.731 (0.678–4.420)
No	78	30.4				
Number of metastatic sites						
≤2	79	28.9	0.312	0.708 (0.361–1.388)	0.344	0.652 (0.269–1.582)
>2	34	39.7				
NLR						
≤3	69	37.4	0.133	1.533 (0.875–2.686)	0.044	2.254 (1.024–4.924)
>3	44	28.7				

Note: DFS, disease-free survival (the time from surgery of the primary tumor to recurrence); HR, hormone-receptor-positive (estrogen receptor (ER) ≥ 10% and/or progesterone receptor (PR) ≥ 10%); NLR, neutrophil-to-lymphocyte ratio.

**Table 5:** Peripheral lymphocyte subsets percentage in MBC patients with low and high baseline NLR.

Peripheral Lymphocyte Subtypes	NLR		*p*
	NLR ≤ 3 (*n* = 77)	NLR > 3 (*n* = 52)	
CD3^+^ T cell	64.0 ± 9.2	58.1 ± 14.1	0.034
CD3^+^CD4^+^ T cell	35.1 ± 8.2	30.4 ± 8.9	0.010
CD3^+^CD8^+^ T cell	27.4 ± 8.7	25.2 ± 10.7	0.141
CD4^+^CD25^+^ T cell	3.5 ± 2.0	3.6 ± 1.9	0.833
CD8^+^CD28^+^ T cell	12.6 ± 6.4	11.9 ± 6.2	0.678
CD8^+^CD28^−^ T cell	20.1 ± 9.6	19.2 ± 8.1	0.944
CD3^−^CD16^+^CD56^+^ NK cell	14.1 ± 8.8	14.7 ± 8.5	0.897
CD19+ B cell	13.7 ± 7.2	13.6 ± 7.6	0.764

Note: MBC, metastatic breast cancer; NLR, neutrophil-to-lymphocyte ratio.

## Data Availability

The data set supporting the results of this article are included within the article and Supplementary Materials. Other datasets used and/or analyzed during the current study are available from the corresponding author on reasonable request.

## Supplementary Materials

The following supporting information can be downloaded at: https://www.mdpi.com/article/10.3390/curroncol29090483/s1, Table S1: Gene list of PredicineCARETM 152-gene panel.
