# Supplementary material for: Prognostic Value of Pretreatment Neutrophil-to-Lymphocyte Ratio in HER2-Positive Metastatic Breast Cancer
Source: Curr Oncol. 2022 Aug 25;29(9):6154–66. doi: 10.3390/curroncol29090483 (PMC9498194; doi:10.3390/curroncol29090483)
Supplement: Supplementary file 1 [file curroncol-29-00483-s001.zip › curroncol-1836592-supplementary.pdf]

Table S1 Gene list of PredicineCARETM 152-gene panel

ABRAXAS1	AKT1	AKT2	AKT3	ALK	APC	AR	ARAF	ARID1A	ATM
ATRX	BAP1	BARD1	BCL2	BRAF	BRCA1	BRCA2	BRIP1	BTK	CCND1
CCND2	CCND3	CCNE1	CCNE2	CD274	CD274 (PD-L1)	CDH1	CDK12	CDK2	CDK4
CDK6	CDKN2A	CHEK1	CHEK2	CTNNB1	CXCR4	CYP2D6	CYP3A4	DAXX	DDR2
E2F1	EGFR	EPCAM	ERBB2	ERBB2 (HER2)	ERBB3	ERCC1	ESR1	EZH2	FANCA
FANCC	FANCF	FANCG	FANCL	FAT1	FBXW7	FEN1	FGFR1	FGFR2	FGFR3
FGFR4	FLT3	FOXA1	FOXL2	FZR1	GEN1	GNA11	GNAQ	GNAS	GSTP1
HNF1A	HOXB13	HRAS	IDH1	IDH2	JAK2	JAK3	KDM6A	KIT	KMT2C
KMT2D	KRAS	MAP2K1	MAP2K2	MAPK1	MAPK3	MDM2	MET	MLH1	MPL
MRE11	MSH2	MSH6	MTOR	MYC	MYCN	MYD88	NBN	NF1	NFE2L2
NOTCH1	NPM1	NRAS	NTRK1	NTRK2	NTRK3	PALB2	PDCD1LG2	PDCD1LG2 (PD-L2)	PDGFRA
PIK3CA	PIK3CB	PIK3R1	PLCG2	PMS2	POLD1	POLE	PPP2R1A	PRKACA	PRKD1
PTEN	PTPN11	RAD50	RAD51	RAD51B	RAD51C	RAD51D	RAD52	RAF1	RB1
RET	RHEB	RHOA	RIT1	RNF43	ROS1	ROS1	SDHB	SMAD4	SMO
SPOP	STAG2	STK11	TERT	TMPRSS2	TP53	TSC1	TSC2	UGT1A1	VHL
XPC	XRCC1								
